# Patients with Mild Cognitive Impairment Display Reduced Auditory Event-Related Delta Oscillatory Responses

**DOI:** 10.1155/2014/268967

**Published:** 2014-03-27

**Authors:** Pınar Kurt, Derya Durusu Emek-Savaş, Kübra Batum, Bilge Turp, Bahar Güntekin, Sibel Karşıdağ, Görsev Gülmen Yener

**Affiliations:** ^1^Brain Dynamics and Multidisciplinary Research Center, Dokuz Eylul University, Balçova, 35340 Izmir, Turkey; ^2^Department of Neurosciences, Dokuz Eylul University, 35340 Izmir, Turkey; ^3^Department of Neurology, Dokuz Eylul University, 35340 Izmir, Turkey; ^4^Department of Psychology, Istanbul Arel University, 34295 Istanbul, Turkey; ^5^Brain Dynamics, Cognition and Complex Systems Research Center Istanbul Kültür University, 35156 Istanbul, Turkey; ^6^Department of Neurology, Maltepe University, 34844 Istanbul, Turkey

## Abstract

*Background.* Event-related oscillations (ERO) may provide a useful tool for the identification of cognitive deficits in mild cognitive impairment (MCI) and Alzheimer's disease (AD). In the present study, we investigate peak-to-peak amplitude of auditory event-related delta oscillations of MCI subjects. *Method.* The study included twenty-two consecutive patients with MCI recruited in neurology clinic and 21 age- and education-matched normal elderly controls. A classical auditory oddball paradigm was used in the experiments. EEG was recorded from F_3_, F_z_, F_4_, C_3_, Cz, C_4_, P_3_, P_z_, P_4_, O_1_, O_z_, and O_2_ locations. The maximum peak-to-peak amplitudes for each subject's averaged delta response (0.5–2.2 Hz) were measured. *Results.* The amplitudes between groups differed significantly at the frontal and mid-centroparietal locations. ANOVA on delta responses revealed a significant effect for *groups* (F_(1.41)_ = 4.84, *P* = 0.033), indicating a larger delta response for healthy controls than MCI subjects. Post hoc comparisons revealed that peak-to-peak delta response was significantly larger for healthy controls than for MCI over electrode sites F_3_, F_z_, F_4_, Cz, C_4_, and P_z_. *Discussion.* Event-related delta frequency band seems to be the most affected oscillatory response in cognitive impairment due to AD. Therefore, it deserves to be investigated as a candidate electrophysiological biomarker in further studies.

## 1. Introduction

The aim of the present study is the analysis of brain oscillations in mild cognitive impairment (MCI). MCI is defined as cognitive deficits that occur before the clinical diagnosis of full dementia syndrome [1]. MCI subjects are considered to have a high risk of dementia, with annual conversion rate to Alzheimer's disease (AD) of 10–18% [2, 3]. The complex neuropathology of MCI that might exhibit the early stages of AD includes plaque- and tangle-formation, neurochemical deficits, cellular injury, inflammation, oxidative stress, changes in genomic activity, synaptic dysfunction, disturbed protein metabolism, and disrupted metabolic homeostasis [4].

Most MCI patients show initial cognitive changes in episodic memory. Besides neuropsychological assessment, neuroimaging techniques (volumetric magnetic resonance imaging (vMRI), functional magnetic resonance imaging (fMRI), fluorodeoxyglucose-positron emission tomography (FDG-PET), and single-photon emission computed tomography (SPECT)) and other candidate biomarkers (cerebrospinal fluid (CSF) concentration of the 42 amino-acid residue amyloid-*β* peptide, tau, and phosphorylated tau protein) are being investigated for clinical diagnosis of MCI [5–8]. Consequently, there is a need to develop a noninvasive, efficient, and low-cost biomarker for diagnosis and monitoring of AD/MCI. Electrophysiological methods may provide such a tool for reflecting brain dynamics and subclinical abnormalities with high temporal resolution; however, they have not yet been widely examined as a diagnostic tool in clinical practice. Recently, there are increasing reports on electroencephalography (EEG) and event-related potentials (ERP) proposing these methods as electrophysiological biomarkers in MCI and AD [9–13]. Higher theta, lower alpha, and increased ratio of alpha3/alpha2 power in MCI patients were reported on quantitative EEG (qEEG) studies [14, 15] The main findings of ERP studies are the prolongation of latency and decrease in amplitude in P300 on frontal regions which were found to be correlated with cognitive dysfunction in MCI patients [16, 17]. Event-related oscillations are the brain's natural responses to sensory or cognitive stimuli, and they provide considerable information about pathological brains, especially in patients with cognitive impairment [18–20] up-to-date clinical studies indicating the importance of brain oscillations.

According to Başar et al., complex and integrative brain functions such as perception, attention, learning, and memory are manifested in the superposition of several oscillations [21]. Several strategies are offered to investigate brain oscillatory activity, including spontaneous EEG and event-related oscillations (ERO). ERO manifests as modification of sensory and cognitive networks elicited upon cognitive task. Further, coherence measurement allows observation of selective connectivity deficit in sensory or cognitive networks [18].

Oscillatory neuroelectric activity has recently been investigated in AD as a candidate electrophysiological biomarker [19, 20, 22–29]. Our group's earlier reports on ERO reported differences between AD subjects and a control group upon the application of oddball paradigm. A previous report related to phase-locking in visual event-related theta oscillations indicated that untreated AD subjects showed reduced phase-locking compared with healthy subjects, and that cholinesterase inhibitor treatment increased phase locking in theta frequency ranges similar to controls [23]. Reduced coherences of delta, theta, and alpha frequency ranges over frontoparietal electrode pairs [22, 26] and higher occipital and parietal sensory visual evoked theta responses [25] in AD patients were also observed. Upon application of the oddball paradigm, decreased amplitude of auditory and visual delta oscillatory responses was observed in AD patients regardless of the cholinergic treatment [24, 26]. Other studies also indicated altered amplitude at slow frequencies in comparison with AD patients over frontal [28] and parietal [30] electrode sites.

In an attempt to extend previous reports in AD, the present study hypothesized that MCI patients would demonstrate lower auditory delta oscillatory responses than healthy elderly controls.

## 2. Methods

### 2.1. Subjects

The study included twenty-two consecutive patients with mild cognitive impairment (MCI group; mean age 74.0 years, age range 60–83) recruited in neurology clinic and 21 age- and education-matched normal elderly controls (mean age 70.3 years, age range 62–85), some of whom were the participants of our other studies [31, 32]. Mean education level was 9.1 years in the MCI group and 9.3 years in the control group (*P* = 0.91) (Table 1). All subjects underwent a detailed neurological evaluation, magnetic resonance imaging (MRI), and cognitive testing that included episodic memory (Öktem verbal memory processes test, ÖVPM, [33], nonverbal memory (WMS-R, visual reproduction test, [34, 35], attention (WMS-R digit span test), orientation, executive functions (Stroop test, [36]; clock drawing test, verbal fluency test), and language (Boston naming test). The cognitively normal participants had intact memory (assessed via verbal memory test ÖVPM) and a clinical dementia rating (CDR, [37]) of zero. The participants diagnosed with MCI exhibited subjective memory complaints, as verified via a family relative, a memory test score that was 1–1.5 SD below the mean age norms, and a CDR of 0.5, not fulfilling dementia criteria (Table 1). Depressive comorbidity was excluded on the basis of a geriatric depression scale score > 11 (GDS, [38]).

The local ethics committee of Dokuz Eylül University approved the study and informed consent was obtained from all subjects or their closest relatives.

Exclusion criteria were abnormal laboratory results indicating other causes of memory disorder, vascular lesions in their MRI, or regular use of antidepressants, neuroleptics, antiepileptic medications, opioids, or beta-blockers. Subjects with vascular lesions on MRI were also excluded from the study. All participants reported normal hearing, and none reported a history of head injury or other neurological or psychiatric disorders.

### 2.2. Stimuli and Paradigm

A classical auditory oddball paradigm was used in the experiments. In the oddball paradigm, audible tones were presented randomly: target tone of 80 dB 1600-Hz occurred approximately 1 : 3 of the time, whereas nontarget tone of 1500-Hz occurred 2 : 3 of the time. The interval between tones varied randomly between 3 and 7 s. The subjects were required to mentally count the number of target tones. During the elicitation period of event-related oscillations, all subjects had displayed sufficient accuracy (error rates < 10%) in mental count of target stimuli, with MCI accuracy being slightly worse than controls.

The auditory stimuli were presented by two loudspeakers and had 16 ms rise time, 50 ms fall time, and 1,000 ms duration.

### 2.3. Electrophysiological Recording

EEG was recorded from F_3_, F_z_, F_4_, C_3_, C_z_, C_4_, TP_7_, TP_8_, P_3_, P_z_, P_4_, O_1_, O_z_, and O_2_ locations according to the international 10–20 system with 30 Ag-AgCl electrodes mounted on an elastic cap (Easy-cap). Additionally, two linked earlobe electrodes (A1+A2) served as references. EOG from the medial upper and lateral orbital rim of the right eye was also registered. For the reference electrodes and EEG recordings, Ag-AgCl electrodes were used. All electrode impedances were less than 10 kΩ. The EEG was amplified by means of a BrainAmp 32-channel DC amplifier with band limits of 0.01–250 Hz. The EEG was digitized on-line with a sampling rate of 500 Hz. The epochs containing artifacts (i.e., eye movement or blink) were rejected by an off-line technique. Subjects' averages and grand averages were calculated for each electrode site and experimental condition.

### 2.4. Power Spectra and Adaptive Digital Filtering

**Figure 1:**
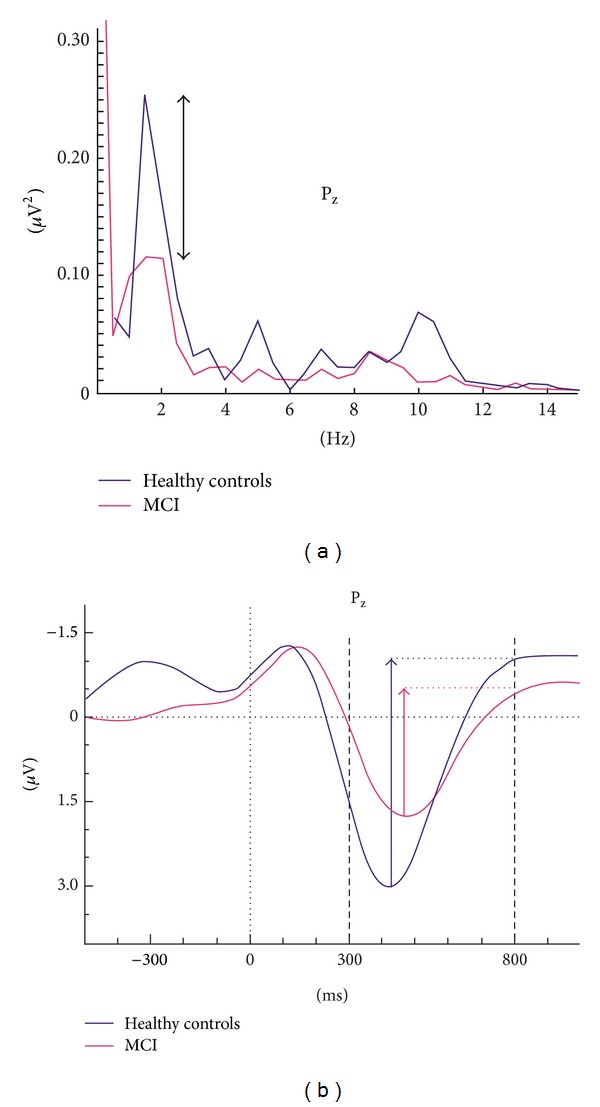
Example of analysis in event-related power spectra and delta response measurements. (a) Superposition of event-related power spectrum of mild cognitive impairment (MCI) (*n* = 22) and healthy subjects (*n* = 21). (b) Measurement of peak-to-peak amplitude.

Prior to analysis of filtered responses, we established the frequency composition of responses by means of event-related power spectra. The event-related power spectra were analyzed by fast-Fourier transform (FFT). There are several techniques for evaluation of power spectra. Bruns showed that several analysis techniques gave similar results [39].  Figure 1(a) shows the power spectrum for grand average of healthy subjects superimposed with that for MCI subjects. The peak-to-peak amplitude measurements were made at the time window of 0–500 ms for both theta (4–7 Hz) and alpha (8–13 Hz) oscillatory responses. It is easily seen that delta peak has a cut-off frequency of 0.5–2.2 Hz. Accordingly, we applied delta band-pass filter of 0.5–2.2 Hz to grand average of ERP (Figure 1(b) shows the grand average of two groups in delta frequency range (0.5–2.2 Hz)). It is immediately seen that the important difference between healthy and MCI subjects lies in the time window between 300 and 800 msec.

It is useful to compute event-related power spectra before deciding the cut-off limits of adaptive digital filters. Depending on the type of cognitive tasks, event-related spectra can show alteration in frequency windows of ERO. Therefore, determination of frequency windows is the most critical part of the ERO analysis in cognitive impairment. Patients can show highly changed frequency windows or frequency shifts. The choice of rigid filters in conventional EEG bands can therefore lead to errors [18].

The power spectrum does not contain temporal information. This means it is not an indicator of phase-locked or nonphase-locked responses upon the onset of stimulation. Conversely, the adaptively filtered event-related delta response (0.5–2.2 Hz) (Figure 1(b)) gives information related to phase-locking and the occurrence of maximal delta response along the time axis [21].

Based on the analysis of power spectra, time-windows for examination were chosen within the range 300–800 msec poststimulus. A human study [40] indicated that corticocortical interplay occurs after sensory-network and hippocampal-cortical network activation of a cortical activation in lateral parietal cortex after approximately 300 msec. The same 300–800 msec time window was therefore chosen for analysis of both target and nontarget responses in the present study.

### 2.5. Statistical Analysis

Statistical analysis used the STATISTICA program. According to cut-off frequencies observed in Figure 1(a), maximum peak-to-peak amplitude responses were analyzed separately for delta target and nontarget (0.5–2.2 Hz), theta target (4–7 Hz), and alpha target (8–13 Hz) frequency bands by means of repeated measures of ANOVA including the between-subjects factor as groups (healthy aged controls, MCI) and the within-subject factors 3 coronal (left, midline, right) × 4 anterior-posterior (frontal, central, parietal, occipital). Greenhouse-Geisser corrected *P* values were taken into consideration. Post hoc analysis was performed using the Bonferroni test.

We also ran another ANOVA to compare the stimulation effect (target versus nontarget) in healthy subjects and in MCI subjects separately. In the analysis, repeated measures of ANOVA included the within-subject factors as stimulation (target versus nontarget) × 4 anterior-posterior (frontal, central, parietal, occipital) × 3 coronal (left, midline, right).

Greenhouse-Geisser corrected *P* values were reported. Post hoc comparisons within group effects were analyzed with paired samples *t*-test. The significance level was set to *P* < 0.05 for all comparisons.

Pearson's correlation analysis was used to determine the correlation between delta responses and the number of errors performed by the subjects during mental count of the target stimuli.

## 3. Results

### 3.1. Behavioral Results

In each measuring session, there were in total 40 auditory target stimuli. Eight of the healthy control subjects counted the target stimulation as 40; three of the healthy subjects made one mistake; and ten of them made more than one mistake while counting the target stimulation. Seven of the MCI patients counted the target stimulation as 40; four of the MCI patients made one mistake; and eleven of them made more than one mistake. Pearson's correlation analysis showed that there were no significant correlations between the number of mistakes and delta oscillatory responses.

### 3.2. Delta Frequency Window

**Figure 2:**
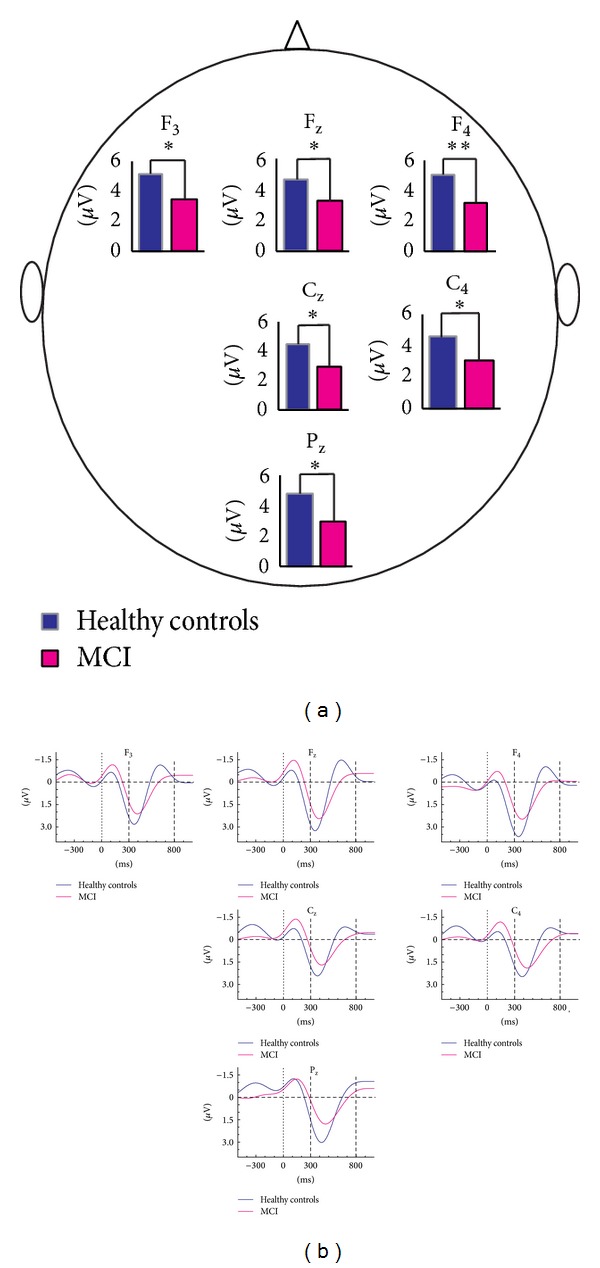
Histograms and grand averages of oscillatory delta (0.5–2.2 Hz) responses. (a) Histograms of mean grand averages of F_3_, F_z_, F_4_, C_z_, C_4_, and P_z_ electrode sites in delta (0.5–2.2 Hz) frequency range for 300–800 msec poststimulus. “∗” represents significant results with *P* < 0.05 and “∗∗” represents significant results with *P* < 0.01. (b) Grand averages of delta (0.5–2.2 Hz) oscillatory responses of healthy controls (*n* = 21) and mild cognitive impairment (MCI) subjects (*n* = 22).

Amplitudes at the frontal and centroparietal locations differed significantly between groups. As seen in Table 2, the peak-to-peak amplitudes at frontal locations were up to 38% larger for healthy controls than for MCI subjects. ANOVA of target delta responses revealed a significant effect for* groups* (F_(1.41)_ = 4.84, *P* = 0.033), with a larger delta response for healthy controls than MCI subjects (see Figure 2(a)). Post hoc comparisons using the Bonferroni test revealed that the peak-to-peak target delta oscillatory response was significantly larger for healthy controls than for MCI over some electrode sites (*P* < 0.01 for F_4_ and *P* < 0.05 for F_3_, F_z_, C_z_, C_4_, and P_z_). ANOVA of delta oscillatory responses revealed a significant effect for* coronal* (F_(1.97)_ = 14.26, *P* = 0.000), indicating increased delta responses over mid- and right-hemisphere recording sites, and* anterior-posterior* (F_(2.08)_ = 6.95, *P* = 0.001), indicating higher delta response over frontal sites compared to central, parietal, and occipital sites.

**Figure 3:**
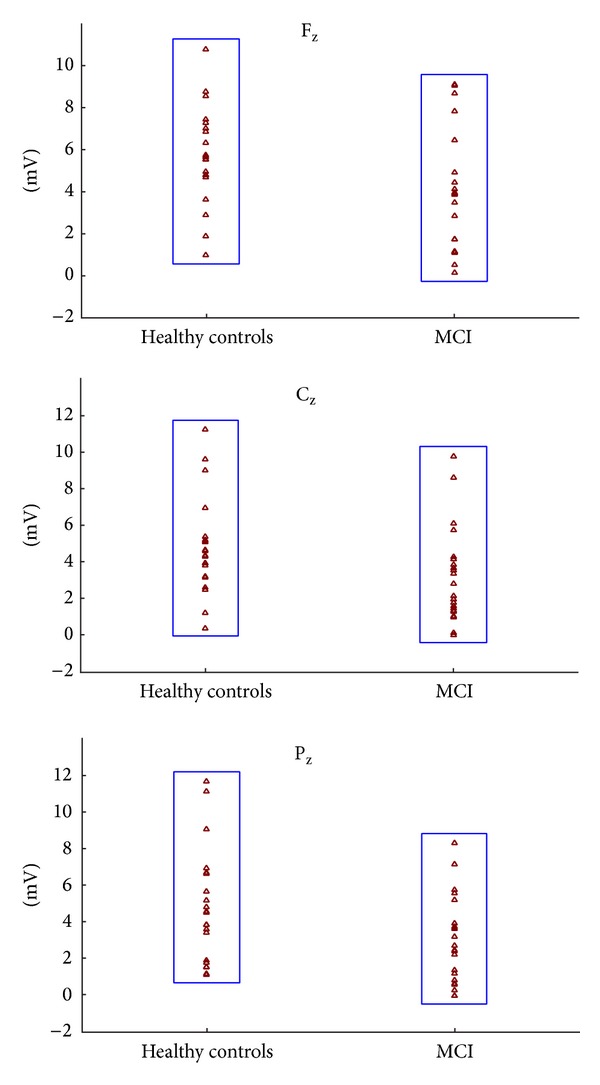
Scattergram of individual delta oscillatory responses of MCI and healthy control subjects on F_z_, C_z_, and P_z_ electrode sites.

The mean (SD) peak-to-peak delta oscillatory response amplitudes of the control group are 4.94 (2.07), 5.78 (2.30), and 5.54 (2.14) *μ*V at locations F_3_, F_z_, and F_4_, respectively. Conversely, MCI subjects showed lower amplitudes of 3.47 (2.17), 3.87 (2.82), and 3.51 (2.23), respectively, at corresponding electrode sites (Table 2).  Figure 3 shows the scattergram of individual delta responses of healthy control subjects and MCI patients.

We evaluated the maximum peak-to-peak amplitudes within delta frequency range of event-related oscillatory responses to auditory target and nontarget signals in MCI and healthy subjects. Figure 2(a) presents all significant results on a topological map. The delta response was larger in healthy controls (indicated by blue line) than in MCI (indicated by purple line) at electrode sites F_3_, F_z_, F_4_, C_z_, C_4_, and P_z_. Compared with MCI subjects, delta response among healthy controls was 30% greater at F_3_, 33% at F_z_, 37% at F_4_, 34% at both C_z_ and C_4_, and 38% at P_z_. In the grand averages of delta oscillatory responses at F_4_, the control group showed larger (*P* < 0.01) amplitude than MCI subjects (Figure 2(b)). No significant differences were recorded at the occipital and temporal locations (Table 2). The delta response to frequent tones was not different between two groups (F_(1.41)_ = 0.372, *P* = 0.54).

In the analysis of targets and nontargets delta (0.5–2.2 Hz) peak-to-peak amplitude revealed that the target stimuli elicited larger delta responses than nontarget stimuli in both healthy controls and MCI patients.

**Figure 4:**
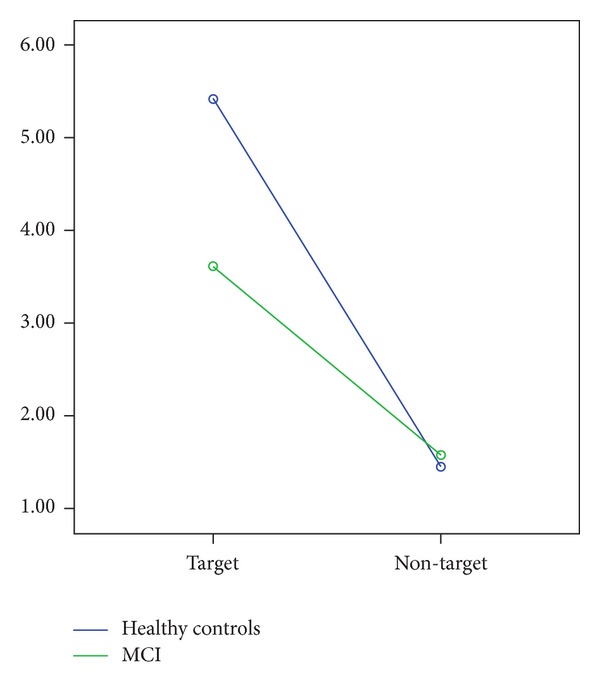
Frontal delta (0.5–2.2 Hz) target and nontarget responses of healthy controls and MCI patients.

Statistical results for 2 group × 2 stimulation × 4 anterior-posterior × 3 coronal were as follows: within-subjects repeated measures of ANOVA revealed a significant difference for stimulation type (F_(1,1)_ = 44.15; *P* = 0.000). The post hoc comparisons revealed that delta response power was significantly greater for target responses than for nontarget responses. Post hoc comparisons showed that there was no significant difference between delta nontarget responses of two groups at any electrode sites (*P* > 0.05). As an example, frontal electrode sites are presented in Figure 4.

### 3.3. Theta Frequency Window

The maximum peak-to-peak theta (4–7 Hz) responses revealed no significant differences between MCI subjects and healthy controls (Figure 4). The ANOVA on theta responses exhibited a significant effect for* anterior-posterior* (F_(3.12)_ = 132.85, *P* = 0.000) indicating that frontal and central theta responses were higher than parietal and occipital theta responses and parietal theta responses were higher than occipital theta responses without group effect.

### 3.4. Alpha Frequency Window

In alpha (8–13 Hz) oscillatory responses, no significant differences were observed between MCI subjects and healthy controls (Figure 4). The ANOVA on alpha responses revealed a significant effect for* anterior-posterior* (F_(3.123)_ = 19.87, *P* = 0.000) indicating that frontal, central, and parietal alpha responses were higher than occipital alpha responses to target stimuli without group effect.

## 4. Discussion

In the present study, a decrease in peak-to-peak amplitude of auditory event-related delta oscillatory responses was recorded in MCI subjects compared to healthy elderly controls. Nonetheless, no differences were found between two groups in the theta, alpha frequency ranges. There are many reports suggesting the diagnostic and/or prognostic role of spontaneous EEG and ERP in AD [9, 12, 13]. In spontaneous EEG, increase of alpha3/alpha2 ratio, higher theta power on frontal regions is suggested to be associated with the atrophy of amygdalo-hippocampal complex [14]. According to Jelic et al., combined alpha and theta relative power were the best predictors of conversion rate from MCI to AD [15]. The oscillatory activity of the brain changes following a simple or cognitive stimuli and the observed responses show differences from those of resting state. Therefore, the observed frequencies of two methods are not comparable to each other. Decreased amplitude and longer latency of P300 are common in MCI patients [16, 17]. Since AD/MCI subjects are characterized by pure cognitive changes, the differences between groups become more evident upon application of cognitive tasks rather than measurement of spontaneous EEG [22]. The result of the present study indicating the decrease in delta oscillatory response in MCI patients seems to be in line with the findings of ERP studies, as delta and theta frequencies are the main components of P300 response. Similar to MCI, among all frequency ranges measured, delta frequency band of event-related oscillatory responses in AD patients showed the most prominent deviation from controls [19, 24, 26, 28, 29].

Earlier studies by our group reported that the peak-to-peak amplitudes of visual delta responses of AD patients were significantly lower at central electrode sites than those of healthy elderly subjects [24]. Upon cognitive stimulation in auditory modality, the differences in delta oscillatory responses between AD patients and healthy controls were observed at frontal, central, and parietal regions [19]. In accordance with these results, Caravaglios et al. reported significant reduction in auditory delta oscillatory responses at frontal electrodes in AD subjects when compared to healthy controls [28]. Polikar et al. used multiresolution wavelet analysis of ERPs to investigate whether EEG can be a reliable electrophysiological biomarker for AD [30]. The authors reported that task-irrelevant novel tones at delta frequency range (1-2 Hz) at the P_z_ electrode provided the most reliable means of distinguishing AD patients from healthy elderly controls. In the present study, MCI subjects showed a 30–38% reduction in auditory target delta responses over the mid-frontocentroparietal regions. In conjunction with the present findings, we may assume that delta oscillatory responses are considerably affected in MCI subjects independent of the stimulus modality. In a recent study of our group, decreased event-related delta oscillatory responses were observed over frontocentroparietal electrode sites in MCI patients upon application of visual oddball paradigm [31]. The two modalities of oddball paradigm revealed slight topographical differences in delta oscillatory responses of MCI and healthy subjects. In the present study, where the peak-to-peak amplitude was examined, the differences were observed at the F_3_, F_z_, F_4_, C_z_, C_4_, and P_z_ electrode sites upon application of auditory oddball paradigm, whereas in visual oddball paradigm, the difference between two groups in delta oscillatory responses was recorded from F_3_, F_4_, C_3_, C_z_, C_4_, P_3_, and P_z_ electrode sites [31]. These findings imply that event-related oscillatory responses are modality independent and mainly reflect the activity of frontal and parietal association areas which are named epicenters of memory-executive function network [41].

Previous ERP studies reported delayed N200 and P300 latency upon application of auditory paradigm among MCI converters [42, 43] and in AD patients [12, 28, 44]. The N200 and P300 responses are interpreted to reflect cognitive level of stimulus processing, and many studies confirmed that P300 response was mainly produced by an oscillatory response in delta frequency range, which is related to focused attention, signal detection, recognition, and decision making [28, 45–48], independent of the modality of the stimulation [47]. It was also shown that the amplitude of delta oscillatory response increases during oddball paradigm and that the major delta response is obtained over frontocentral region upon auditory stimuli [49–51]. In line with these previous studies, in our study, both groups of subjects revealed higher delta responses to target stimuli than nontarget stimuli and the delta oscillatory responses to frequent tones of MCI patients did not differ from those of healthy control subjects. These findings emphasize the role of delta oscillatory activity in attentional and/or cognitive processes.

In the present study, theta and alpha oscillatory responses did not show any differences between MCI and healthy subjects. In our groups' previous studies MCI, AD, and healthy controls did not differ in the peak-to-peak amplitude measurement values of event-related theta and alpha responses [22, 23]. The phase-locking and coherence measurements of theta and alpha oscillations are suggested to be analyzed in future studies.

Similar to previous reports in AD patients, the results of this study indicate that auditory delta oscillatory responses of MCI patients are decreased at frontal, central, and parietal locations. Many studies concluded that episodic memory impairment is frequently the first symptom to appear in MCI, being a prodromal form of AD [1, 2, 52]. However, disruption of other cognitive abilities such as selective attention and executive functions is also manifested in the very early stages of the disease [2, 53] as a result of cholinergic deficiency [54]. Keeping in mind that delta oscillatory responses are related to attention and decision making, the decrease in delta amplitude of MCI patients observed in the present study might also indicate attentional deficits and executive dysfunction among these patients.

**Figure 5:**
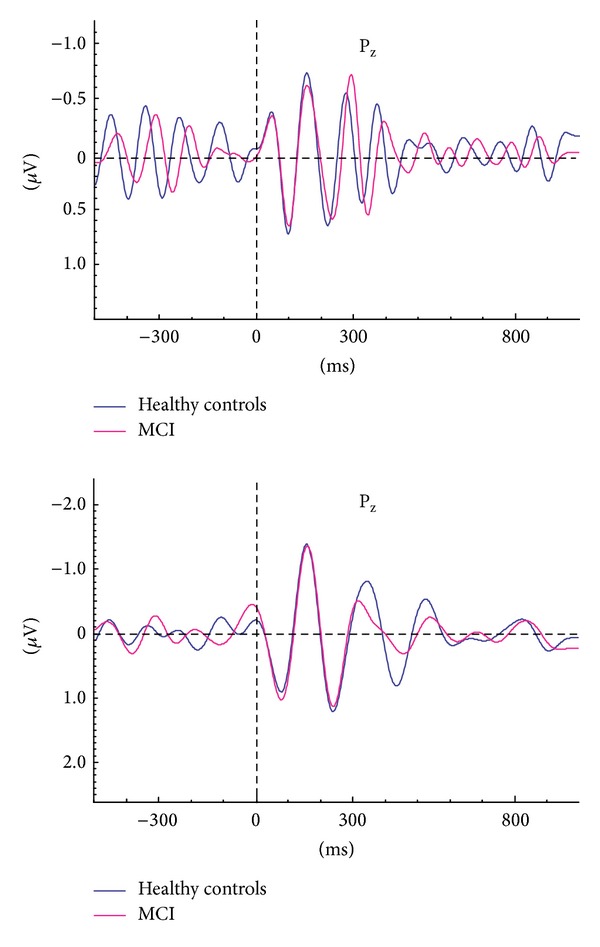
Grand averages of alpha (8–13 Hz) and theta (4–7 Hz) oscillatory responses of healthy control subjects (*n* = 22) and MCI (*n* = 21) patients.

The neuropathological and structural changes in MCI show similar pattern to mild AD [4]. Currently, structural, metabolic, and CSF biomarkers are becoming widely used in the diagnosis and prognosis of AD/MCI (Figure 5). In order to develop neurophysiological biomarkers for the diagnosis and prognosis of AD, the combined use of validated markers with electrophysiological methods has been proposed [18].

Event-related oscillations may reflect cognitive changes in neuropsychiatric disorders. Recently, our group analyzed ERO activity in AD subjects; the findings included several methods, such as peak-to-peak amplitude measurements, phase locking, and degree of coherence [18, 19].

Yener et al. examined the peak amplitudes of event-related oscillatory responses within specific frequency ranges in AD subjects (both untreated and those on cholinesterase treatment) compared to those of healthy elderly controls [23]. They found that de novo AD patients showed reduced theta phase-locking oscillatory activity at left-frontal electrode sites compared to both healthy controls and cholinergically medicated AD subjects. However, reduced oscillatory delta response was observed in all AD patients irrespective of the cholinergic therapy [24, 25]. Event-related coherence studies in the AD group also showed consistent decrease of delta, theta, and alpha coherences between frontal and all other regions of the brain upon visual oddball paradigm.

**Figure 6:**
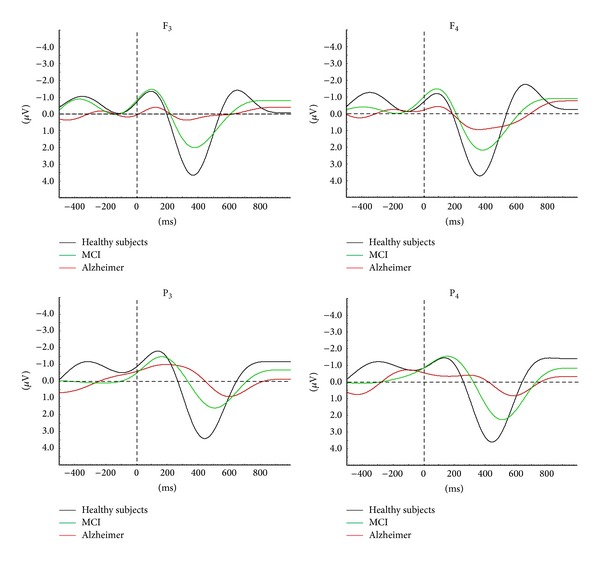
MCI and AD continuity is prominent in auditory event-related delta oscillatory activity. Results show gradually decreasing delta amplitude and increasing delta peak latency among healthy elderly subjects, MCI, and mild-stage Alzheimer subjects. MCI: mild cognitive impairment; AD: Alzheimer's disease [18].

In agreement with the abovementioned series of ERO studies in AD subjects [19, 22, 28, 29], the present study found reduced delta event-related oscillatory responses in MCI patients. In combination, these results point to MCI and AD continuity by auditory event-related delta oscillatory activity. A recent report showed gradually declining amplitude of delta oscillatory responses across healthy elderly subjects > MCI > mild-stage Alzheimer subjects (Figure 6) [18].

As shown in the present study, delta responses are also reduced in MCI, although to a lesser degree than AD, and these electrophysiological findings indicate the continuity between MCI and AD.

## 5. Conclusion

Electrophysiological methods provide noninvasive, rapid, and replicable method for assessing age-related and disease-related neurophysiologic changes. In search of electrophysiological biomarkers for cognitive impairment, changes in a unique oscillatory component should be considered as only one of the components among other brain oscillatory responses and possible connectivity deficits. Reduction of target delta oscillatory response in either visual or auditory modality is a candidate parameter for differentiating MCI from healthy controls. As proposed by Yener and Başar [19, 20], several steps are required to achieve a complete description of an ensemble of neurophysiological markers in a disease, including analysis of oscillatory responses in several stimulation modalities encompassing tasks and different sensory stimulation. Therefore, the results of the present study contribute to the completion of these electrophysiological biomarkers. In order to validate electrophysiological methods as potential neurophysiological biomarkers in AD/MCI, further studies should be conducted using multimodal techniques. In addition, greater methodological standardization and the development of normative age-referenced databases of peak-to-peak amplitude values for event-related delta oscillatory responses are required in further studies.

## Figures and Tables

**Table 1:** Demographic and neuropsychological details of study subjects.

	Controls (*N* = 21) Mean (SD)	MCI (*N* = 22) Mean (SD)	*P *
Age (SD)	70.3 (6.5)	74.0 (7.0)	0.08^a^
Education (SD)	9.3 (5.0)	9.1 (5.1)	0.91^a^
Gender (M/F)	8/13	13/9	0.17^b^
MMSE	28.7 (1.9)	24.9 (3.0)	0.000^a^
ÖVPMT (short-term memory)	104.5 (13.2)	68.7 (13.2)	0.000^a^
ÖVPMT (free recall)	12.4 (1.7)	4.8 (3.2)	0.000^a^
ÖVPMT (recognition)	14.8 (0.5)	13.7 (1.3)	0.002^a^
Digit span forward	4.8 (1.1)	4.9 (0.88)	0.65^a^
Digit span backward	3.8 (1.3)	3.3 (0.7)	0.17^a^
Verbal fluency (animal list)	21.3 (4.3)	16.7 (4.5)	0.003^a^
Stroop interference (sec)	54.7 (14.1)	85.3 (46.8)	0.013^a^

MCI: mild cognitive impairment; MMSE: mini-mental state examination; ÖVPMT: Öktem Verbal Processes Memory Test; GDS: Geriatric Depression Scale; sec: seconds; SD: standard deviation; M: male; F: female; ^a^independent sample *t*-test; ^b^chi-square.

**Table 2:** Mean, standard deviation, and *P* values of maximum peak-to-peak amplitudes of auditory target delta (0.5–2.2 Hz) oscillatory activity in healthy elderly controls and mild cognitive impairment (MCI) subjects.

Location	Healthy controls (*n* = 21)	MCI (*n* = 22)	*P* value
	Mean value *μ*V (SD)	Mean value *μ*V (SD)	
F_3_	**4.94 (2.07)**	**3.47 (2.17)**	**0.029**
F_z_	**5.78 (2.30)**	**3.87 (2.82)**	**0.019**
F_4_	**5.54 (2.14)**	**3.51 (2.23)**	**0.004**
C_3_	3.86 (2.25)	2.83 (1.92)	0.114
C_z_	**4.81 (2.65)**	**3.17 (2.55)**	**0.044**
C_4_	**4.72 (2.22)**	**3.12 (1.92)**	**0.014**
TP_7_	2.33 (1.47)	2.04 (1.44)	0.523
TP_8_	3.62 (1.71)	2.70 (1.39)	0.058
P_3_	4.16 (2.81)	3.04 (2.21)	0.149
P_z_	**4.92 (3.08)**	**3.04 (2.34)**	**0.03**
P_4_	5.05 (3.01)	3.66 (2.33)	0.104
O_1_	3.99 (2.64)	2.81 (1.96)	0.099
O_z_	3.85 (2.65)	3.01 (2.01)	0.246
O_2_	3.85 (2.43)	3.08 (1.98)	0.261

Statistically significant (*P* < 0.05) results are indicated in bold style.
